# Highly ordered CaO from cuttlefish bone calcination for the efficient adsorption of methylene blue from water

**DOI:** 10.3389/fchem.2023.1132464

**Published:** 2023-02-16

**Authors:** Uroosa Tagar, Maurizio Volpe, Antonio Messineo, Roberto Volpe

**Affiliations:** ^1^ School of Engineering and Materials Science, Queen Mary University of London, London, United Kingdom; ^2^ Faculty of Engineering and Architecture, University of Enna Kore, Cittadella Universitaria, Enna, Italy

**Keywords:** calcium oxide (CaO), calcination, cuttlefish bone, methylene blue (dye), water remediation

## Abstract

The aim of this study is to synthesize cheap and highly ordered CaO from cuttlefish bone (CFB) as a green alternative to conventional adsorbents such as activated carbon. This study focuses on the synthesis of highly ordered CaO via calcination of CFB, at two different temperatures (900 and 1000°C) and two holding times (0.5 and 1 h), as a potential green route for water remediation. The as-prepared highly ordered CaO was tested as an adsorbent using methylene blue (MB) as a model compound for dye contaminants in water. Different CaO adsorbent doses (0.05, 0.2, 0.4, and 0.6 g) were used, keeping the MB concentration fixed at 10 mg/L. The morphology and crystalline structure of the CFB before and after calcination was characterized *via* scanning electron microscope (SEM) and X-ray diffraction (XRD) analyses, while the thermal behavior and surface functionalities were characterized by thermogravimetric analysis (TGA) and Fourier transform infrared (FTIR) spectroscopy, respectively. Adsorption experiments using different doses of CaO synthesized at 900°C for 0.5 h showed an MB removal efficiency as high as 98% by weight using 0.4 g (adsorbent)/L(solution). Two different adsorption models, the Langmuir adsorption model and the Freundlich adsorption model, along with pseudo-first-order and pseudo-second-order kinetic models, were studied to correlate the adsorption data. The removal of MB *via* highly ordered CaO adsorption was better modeled by the Langmuir adsorption isotherm giving (R^2^ =0.93), thus proving a monolayer adsorption mechanism following pseudo-second-order kinetics (R^2^= 0.98), confirming that chemisorption reaction occurs between the MB dye molecule and CaO.

## Graphical Abstract

**Figure:**
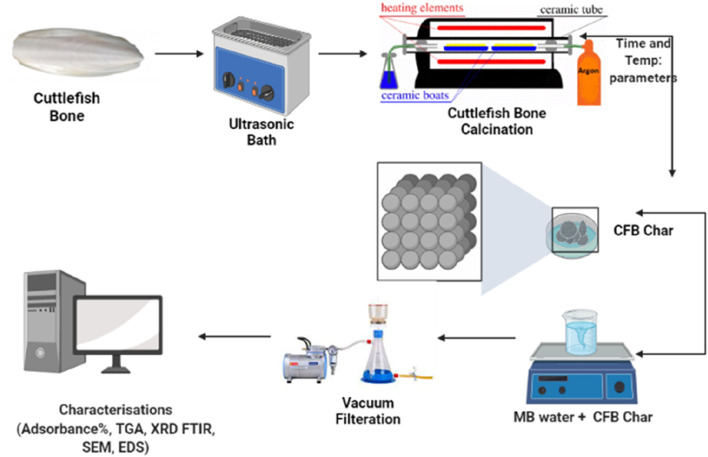
Schematic illustration of the proposed work.

## 1 Introduction

Severe water pollution and increasing *per capita* clean water demand are stressing our natural water resources (Tripathi and Rawat Ranjan, 2015; Sahle-Demessie et al., 2019). On 25 September 2015, member states of the United Nations adopted the 2030 agenda for sustainable development, which aims at ensuring access to clean water, sanitation, and sustainable water management for all (UNICEF and WHO, 2018). Suspended particles, color, pH, temperature, and chemical oxygen demand (COD) concentrations are some of the problematic characteristics of wastewater. Dye wastewater that is simply dumped in the environment is a major environmental risk (Vimonses et al., 2009). Textile dyeing is the second-largest polluter of water worldwide, where the production processes generate large volumes of wastewater containing color that eventually contaminates groundwater and freshwater streams, especially where non-optimal water management is in place, such as in less developed countries (Mostafa, 2015). According to recent data, approximately 100 thousand commercially dyed products with over 7 × 10^5^ tons of synthetic dyes are annually produced worldwide (Drumond Chequer et al., 2013), and 2%–20% of dyes (Carmen and Daniel, 2012) used in industrial applications have been released into the aquatic environment. The textile industry is the principal responsible industry for dye-based water pollution, followed by the printing paper, paint, and leather production industries (Hamad and Idrus 2022).

Water treatment technologies, including water desalination and purification, are of great interest. A variety of physical, chemical, and biological methods, such as adsorption, coagulation, membrane filtration, precipitation, reverse osmosis, and oxidation–ozonation, have been developed for wastewater treatment (Lee et al., 2011). Reverse osmosis and thermal treatments are widely used by large-scale plants, which consume a large amount of energy and require skilled labor and highly efficient machines, which makes the technology ill-suited to developing countries (Yang et al., 2013). Adsorption offers a potentially lower cost, simple operation, and efficient alternative to water filtration, particularly suitable to less developed areas of the world. It is the simplest method, has a low operational cost, is easy to handle, is highly efficient, and has an easy regenerative technique (Bayomie et al., 2020). Diverse adsorbents are available to treat wastewater, such as activated carbons, zeolites, and graphene (Nigiz, 2019); however, these materials have limited applicability owing to their high cost and low reusability (Crini, 2006; Gupta and Suhas., 2009; Chikri et al., 2020). In recent years, several low-cost and widely available adsorbents from agricultural and marine wastes have been proposed for efficient dye removal (Foo and Hameed, 2010). Thermal and chemical treatments are used to optimize the adsorption characteristics and performance of this biomass. This biomass includes Fox and Brazil nutshells (de Oliveira Brito et al., 2010; Kumar and Jena, 2016), fava bean peel (Bayomie et al., 2020), activated carbon prepared from coconut shell (Ahmed et al., 2017), tamarind and yellow passion fruit shell (Jacques et al., 2007), hen feathers (Chowdhury and Saha, 2012), palm and phoenix tree leaves (Han et al., 2007; Belala et al., 2011), pomelo leaves (Hameed et al., 2008), rice husk (Shen et al., 2014), banana peel (Amel et al., 2012), and many more.

CaO-based adsorbents have been greatly used for different applications. Quicklime (CaO) and calcium hydroxide [Ca(OH)_2_] have been used to treat biological organic wastes for more than 100 years. Calcium hydroxide is an alkaline compound that can create pH levels as high as 12.4. At pH levels greater than 12 at increased temperatures, cell membranes of harmful pathogens are destroyed (Sawai, 2011). Extensive investigations on calcium-based photocatalysts have been undertaken, for example, on CaMgO_2_ nanoflakes (Karuppusamy et al., 2021), [Ca(OH)_2_] for the effective removal of indigo carmine dye (Ramesh et al., 2017), and calcium oxide (CaO) for MB removal (Rameshwar and Dileep Kumar, 2014). Furthermore, Ca(OH)_2_ is also used for the lignocellulosic pre-treatments for enzymatic hydrolysis of sugarcane bagasse (Chang et al., 2017). Ca(OH)_2_ pre-treatment is safer to operate than NaOH pre-treatment due to its weaker caustic feature. (Nie et al., 2019) conducted a study for the simultaneous removal of bisphenol-A (BPA) and phosphate (P) using the combination of Ca(OH)_2_ and peroxymonosulfate (PMS). In addition, Ca(OH)_2_ can react with P to form insoluble precipitates, and it was successfully employed to remove P in wastewater (Nie et al., 2019). CaO-based sorbent pellets capture CO_2_ from flue gas. Synthetic calcium sources are expensive, such as the unit price of calcium acetate is over 20 times that of natural limestone to produce hydrated lime. CaO is also used for biodiesel production due to its high basicity, lower toxicity, high stability, easy handling, and low cost (Acosta et al., 2020).

Calcium-based materials are mostly synthesized from chicken eggshell waste (Witoon, 2011; Mohamed et al., 2021), shrimp shells (Gedda et al., 2015), and other marine molluscan shell waste (Anand et al., 2021; Vijai Anand et al., 2021). Calcination is a high-temperature, chemical-free breakdown method for converting marine-sourced CaCO_3_ into CaO (Haryati et al., 2021). Calcination temperature is the key factor in the process and generally depends on the nature of CaCO_3_ or other compounds presented before calcination (Tlili et al., 2021). The temperature-dependent changes in calcium oxide’s properties and phase transitions are also fascinating to learn about. According to Soisuwan et al. (2014), the best calcination temperature for calcium carbonate from oyster shells, mussel shells, and CFB into calcium oxide is 850°C. Suwannasingha et al. (2022) analyzed the effects of calcination temperature (550°C, 700°C, and 900°C) on characteristics and elemental composition of calcium oxide derived from oysters, green mussels, blue swimming crabs, and CFB (Tlili et al., 2021).

**FIGURE 1:**
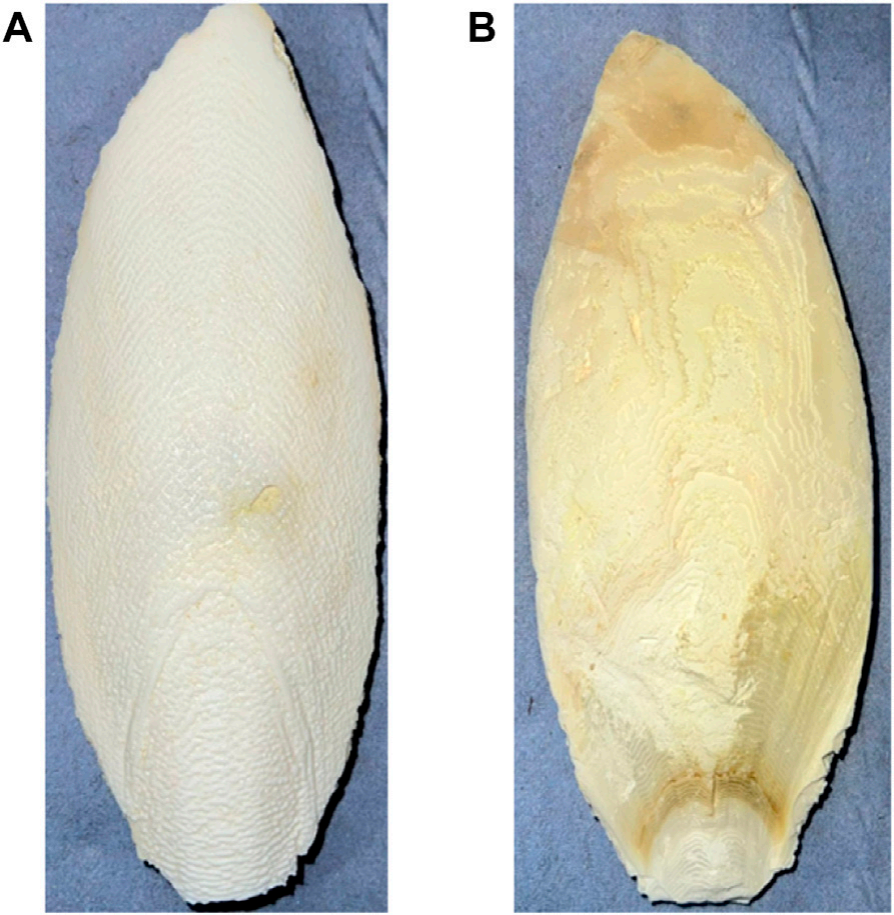
CFB used for this study: **(A)** front side and **(B)** back side.

In this work, we propose a highly ordered CaO-based adsorbent synthesized from CFB, a natural biowaste, using two different temperatures (900°C and 1000°C) for two different holding times (0.5 h and 1.0 h). As per our literature survey, the 1000°C temperature used for CaO synthesis from CFB is the highest temperature used for CFB. It is very interesting to see what morphological changes are induced in highly stable CaO at higher temperatures. Increased calcination temperatures encourage faster calcination rates since they hasten the conversion of CaCO_3_ to CaO and result in shorter working times. Figure 1 shows the CFB used in this work—a brittle skeleton structure found in all members of the cephalopod family, which is made of two main components: (a) dorsal shield and (b) lamellar matrix (Vibhatabandhu, 2015). Chemically, CFB is mainly composed of aragonite (85%), a carbonate mineral. CaCO_3_ can exist in three polymorphic forms, which, in the order of their usual stabilities, are calcite, aragonite, and vaterite (Simkiss, 1932).

The as-prepared CaO was tested as an adsorbent for the efficient degradation of MB dye from water in an attempt to offer a cheaper and more sustainable alternative to synthetic adsorbents. The effect of CaO biosorbent dosage rate (g) (0.05 g, 0.2 g, 0.4 g, and 0.6 g) and reaction time (min) varied from 10 min to 24 h on standard MB concentration 10 (mg/L) and was studied using a UV–visible spectrophotometer. The kinetic study of highly ordered CaO was carried out using two adsorption isotherm models, Langmuir and Freundlich adsorption isotherms, and the analyzed data were fitted using pseudo-first-order and second-order kinetic models.

## 2 Materials and methods

### 2.1 Materials

CFB was purchased from Britten and James online store at Amazon.com, in average 5.5–8 inches cuttlebone pieces. The dorsal shield was easily removed using a retractable knife, while the lamellae portion was cut into average 1-inch small cubes that were employed for the synthesis of CaO. The small cubes were first washed with ultra-pure water with a resistivity of 18.2 MΩ cm and a purity level of 100%. Then, the bone cubes were sonicated at 25°C for 30 min in ethanol. After sonication, the CFB cubes were dried in a Heratherm oven (Thermo Fisher Scientific) at 80°C for 4 h. As CFB was sonicated in ethanol, so the 4 h time duration was sufficient. For CFB calcination, cleaned and dried CFB cubes were crushed using mortar and pestle to obtain CFB powder (CFBP). MB dye, pure certified and with CAS number 7220-79-3, was obtained from Thermo Fisher Scientific Ltd., United Kingdom. Ultra-pure water was used for MB solution preparation for the whole experiment.

### 2.2 CFB calcination

Before calcination, the moisture content of CFBP was calculated by oven drying at 105°C for 2 h. Dried CFBP was calcined at 10°C/min heating rate, to 900 and 1000°C peak temperatures for 0.5 and 1 h residence times, using a fixed bed lab-scale furnace (type LTF 12/50/300 manufactured by Lenton thermal designs) under a continuous argon gas flow of 1 L/min. The thermal decomposition reaction of CaCO_3_ and the corresponding variation of hentalpy are reported as follows (Battistella et al., 2012):

$$CaCO_{3\;\left(s\right)}\to CaO_{\left(s\right)}+CO_{2\;\left(g\right)\;}\Delta H=178\frac{kJ}{mol}.$$ (1)

After calcination, the furnace was left to cool down to room temperature, and samples were stored in a desiccator before analytical characterizations.

### 2.3 Analytical characterizations

Weight loss of CFBP concerning temperature was investigated using a TA Instruments Q500 thermo-gravimetric analyzer. The samples were ramped up to 900°C with a holding time of 20 min and a heating rate of 10°C/min with a constant nitrogen gas flow at 60 ml/min. The X-Ray diffraction (XRD) patterns of washed CFBP and calcined CFBP were carried out by a Siemens D5000 X-Ray powder diffractometer using Cu Ka radiation (λ= 0.154 nm) in the 2θ range of 5–70°. The Fourier transform infrared (FTIR) spectra of the CFBP and calcined CFBP were collected on a Bruker Tensor 27 FTIR spectrometer in 400–4000 cm^-1^. The analysis was run in attenuated total reflection (ATR) mode with 16 scans per minute. The surface morphology and energy-dispersive x-ray spectroscopy (EDS) analyses of the CFBP before and after calcination was observed using an analytical scanning electron microscope (SEM) manufactured by Joel. The samples were gold-coated (4-nm coating thickness) using Agar Auto sputter coater and analyzed at different magnifications mentioned in SEM images, applying 5 kV voltage. Brunauer–Emmett–Teller (BET) surface area and pore size distribution were calculated using NOVA 4200, and the samples were degassed for 24 h at the same machine. Ultraviolet-visible (UV–Vis) adsorption analysis was carried out using Perkin Elmer Lambda 35 UV–Vis spectrometry in the range of 400–700 nm wavelength for methylene blue studies.

### 2.4 Methylene blue stock solution preparation and analysis

A standard stock solution (100 mg/L) of MB was prepared by dissolving 100.0 ± 0.1 mg of MB dye in ultra-pure water using a 1000-mL class “A” volumetric flask. A 10-mg/L MB working solution was prepared by dilution of the standard stock solution. UV–Vis adsorption at (λmax) of 663 nm was used to monitor the MB concentration, and determination of the extinction coefficient ɛ = 74028 M^−1^ cm^−1^ using the Beer–Lambert law at 663 nm is reported as follows:

A=ɛ×l×c, (2)

where A= absorbance, ɛ = molar absorptivity (M^−1^ cm^−1^), l = optical path length (cm), and c= molar concentration (M).

### 2.5 Adsorption experiments

Different MB concentrations have been reported in the literature for MB adsorption experiments. Following the trend, we selected an MB concentration of 10 mg/L for our studies. All the experiments were carried out in 50-mL class “A” volumetric flasks, and different adsorbent loadings (0.05, 0.2, 0.4, and 0.6 g) of calcined CFBP in solutions were left stirring at 200 rpm for a contact time of 10 min 20 min and between 0.5 and 24 h. Optimization of the parameters affecting adsorption was carried out by varying one parameter at a time while keeping others the same, including dye concentration, contact time, and adsorbent dosage. After the predetermined contact time was passed, the solutions were filtered using 13-mm non-sterile polytetrafluoroethylene (PTFE) membrane syringe filters with a filter pore size of 0.2 µm. The amount of MB adsorbed at equilibrium per unit mass of biosorbent (qe) was calculated using

$$qe=\frac{=\left(C_{0}-C_{e}\right)\times V}{m}.$$ (3)

The calculation of the MB rate percentage of adsorption also called removal% (R %) was evaluated using

$$R\%=\frac{=\left(C_{0}-C_{e}\right)}{C_{0}}\times 100,$$ (4)

where C_o_ is the initial MB concentration (mg/L), Ce is the equilibrium concentration of the adsorbate (mg/L) in solution, V is the volume of solution (L), m is the mass of calcined cuttlefish bone (g), and qe is the methylene blue quantity adsorbed at equilibrium (mg/g). Tests were normally run in triplicates, and any results beyond 2% error from average were discarded. To get an insight into the adsorption mechanism, the data were fitted using two adsorption isotherms models, the Langmuir and Freundlich ones. The Langmuir adsorption isotherm describes gas–solid-phase adsorption and assumes monolayer adsorption. The Freundlich empirical model can be applied to multilayer adsorption, with non-uniform distribution of adsorption heat and affinities over the heterogeneous surface.

## 3 Results and discussion

### 3.1 SEM and EDX analysis of CFBP and calcined CFBP

**FIGURE 2:**
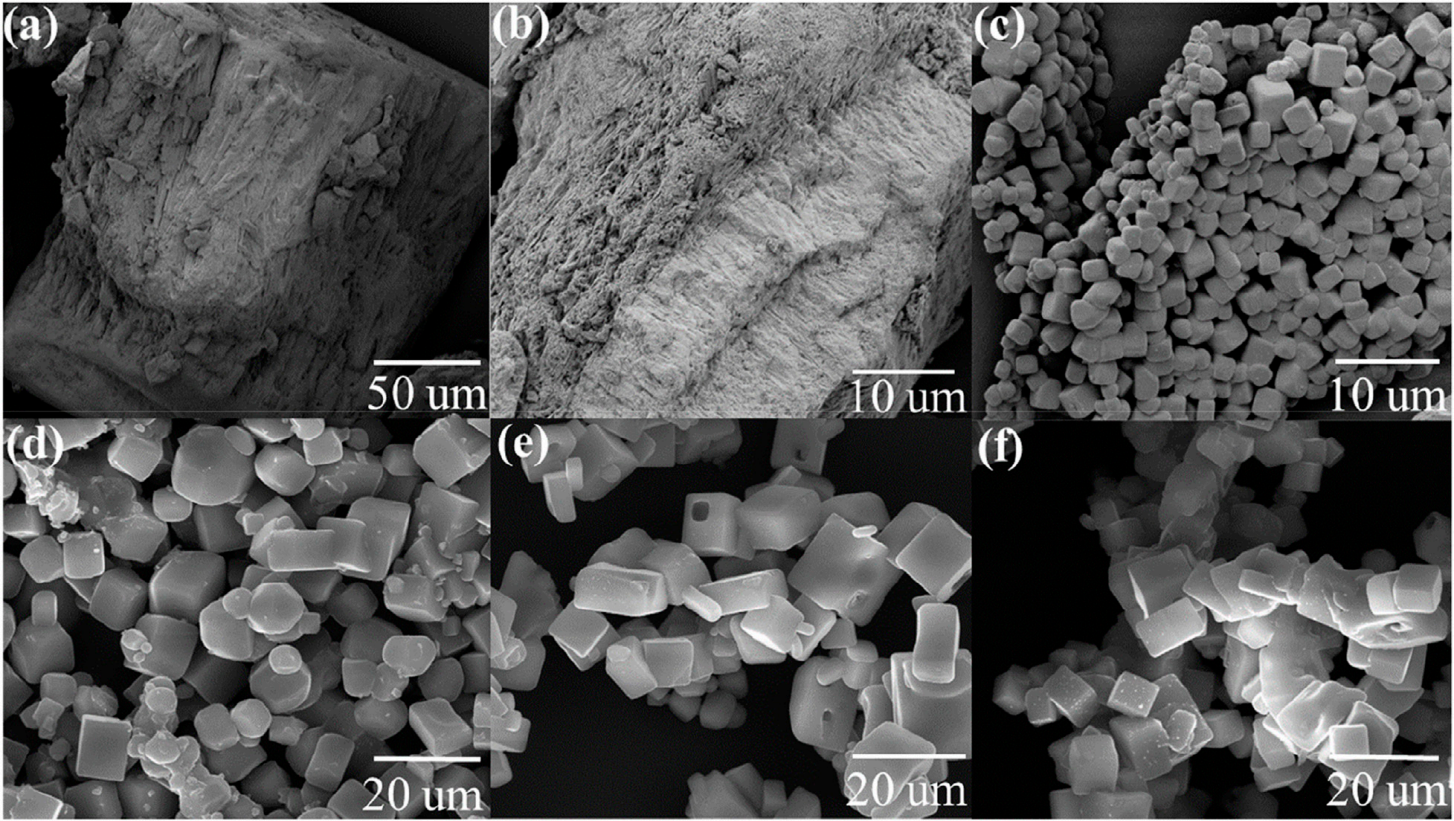
SEM images of cuttlefish bone: **(A)** pure washed CFB top dorsal part before calcination, **(B)** washed CFBP lamellae part before calcination, **(C)** washed CFBP calcined at 900°C 0.5 h, **(D)** washed CFBP calcined at 900°C 1 h, **(E)** washed CFBP calcined at 1000°C for 0.5 h, and **(F)** washed CFBP calcined at 1000°C 1 h.

To evaluate the morphological changes by heat treatment, the CFBP’s morphology was examined using SEM before and after calcination. Figure 2 (a) washed CFB top dorsal part before calcination, (b) washed CFBP lamellae part before calcination, (c) washed CFBP calcined at 900°C for 30 min, (d) washed CFBP calcined at 900°C for 60 min, (e) washed CFBP calcined at 1000°C for 30 min, and (f) washed CFBP calcined at 1000°C for 60 min.

Raw CFBP shows an irregular surface structure that was fully converted into a solid cubicle structure upon calcination at 900°C, 0.5 h, as can be seen in Figure 2C. Similarly, Khan et al. (2018) studied the green calcium hydroxide nano-plates derived from waste oyster shells. They showed that calcination of oyster shells at 900°C for 2 h produced peanut-shaped grains forming a compact structure. Furthermore, longer times of calcination at 900°C (1.0 h) showed an influence on cubical structures CaO, started agglomeration, and appeared less regular in shape and size, as shown in Figure 2C. The increase in the calcination temperature promoted a general agglomeration of the individual particles. At the calcination temperature of 1000°C, the regular cubical shape of CaO is no longer retained, possibly leading to a smaller surface area and porosity of the samples.

There have been lots of investigations carried out on the influence of temperature and heating rates on different marine wastes. Saraidin (2014) analyzed the influence of temperature and heating rates on the calcination of waste cockle shells to calcium oxides. The study shows that the cockle shell’s structure changed after 700°C, but the structure of CaO was not uniform as partial calcination occurs at a lower temperature. At higher temperatures of 800°C and 900°C, the curves were smoother and had a more uniform surface. The samples showed more energy at higher temperatures hastening their kinetic motion and the calcination process. Furthermore, Suwannasingha et al. (2022) studied the influence of temperature and heating rates on the calcination of four different marine waste shells to CaO. They used shells of oysters, green mussels, blue swimming crabs, and cuttlefish for calcination and analyzed the effects of calcination temperature (550°C, 700°C, and 900°C) on characteristics and elemental composition of CaO derived from each type of shell. At 550°C, the color of the green mussel shell, blue swimming crab shell, cuttlebone, and oyster shell changed significantly from the original color to dark grey or black color with the lowest L* values. Compared to samples that were calcined at 550°C, all samples' L* values dramatically increased at 700°C. A similar L* increment was seen at 900°C (Suwannasingha et al., 2022). The macroscopic changes described are caused by changes in the crystalline structure of the bone. When bones are treated at higher temperatures, they initially lose strength at 600°C while regaining some strength at elevated temperatures such as 850°C and above. Van Strydonck et al. (2005) and Vanthana Sree et al. (2020) discussed the effect of calcination temperature on calcium oxide synthesis from eggshell powder. They have discussed that at lower temperatures such as 700°C (1 h) and 700°C (2 h), powders are in black and dark grey colors due to incomplete CO_2_ emission, whereas at 700°C (3 h), slightly grey powder was produced. At 800°C (1 h) and 800°C (2 h) calcination, the product obtained was white and partially solid but contained tiny solid particles like eggshell powder. Samples from 800°C (3 h) and 900°C (1 h) resulted in fine CaO nanoparticles with 46% weight loss due to complete CO_2_ removal from CaCO_3_, but the morphological structure of these conversions was not identical and appeared to be clusters of flack like structure. These studies lack a detailed discussion of morphological changes occurring at higher temperatures, such as 1000°C, with an increased holding time.

**FIGURE 3:**
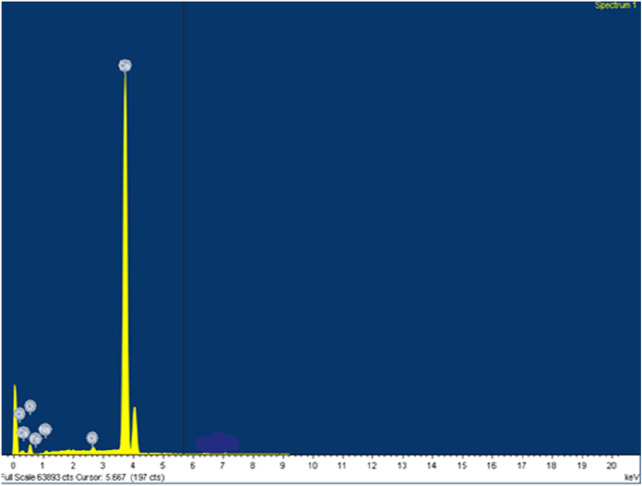
EDS image of calcined cuttlefish bone.

Natural seashells display typical layered structures. Similarly, raw CFB shows irregular surfaces, as shown in Figure 2. By calcination of the CFBP at 900°C for 0.5 h, the layered architecture was changed to a stable and highly ordered cubicle structure. The size and morphology of the CaO nanoparticles of CFB waste were confirmed by SEM measurements, which showed that the particles were well dispersed with cubicle morphology (Anand et al., 2021; Vijai Anand et al., 2021). A balance between the reaction temperature and reaction dwell time is desirable in the calcination process. On the one hand, short residence times could lead to a partial CaCO_3_ conversion into CaO; on the other hand, long residence times could result in the shrinkage of sample volume by closing the pores and disallowing the release of CO_2_. To increase the system efficiency, balanced reaction parameters must be set for the complete conversion of CaCO_3_. Compared to the literature, we achieved the full conversion of CaCO_3_ into CaO with very less holding time (0.5 h). This helped in saving a huge amount of energy on a larger scale. The second advantage of using CFB at 900°C (0.5 h) was that a solid, regular, and identical cubicle structure of CaO was achieved. Figure 3 shows EDS analysis confirming calcium and oxygen weight mass composition of 71 wt% and 29 wt%, respectively, corresponding to a pure sample of CaO.

### 3.2 Surface area and particle size distribution of CaO synthesized at different temperatures

**FIGURE 4:**
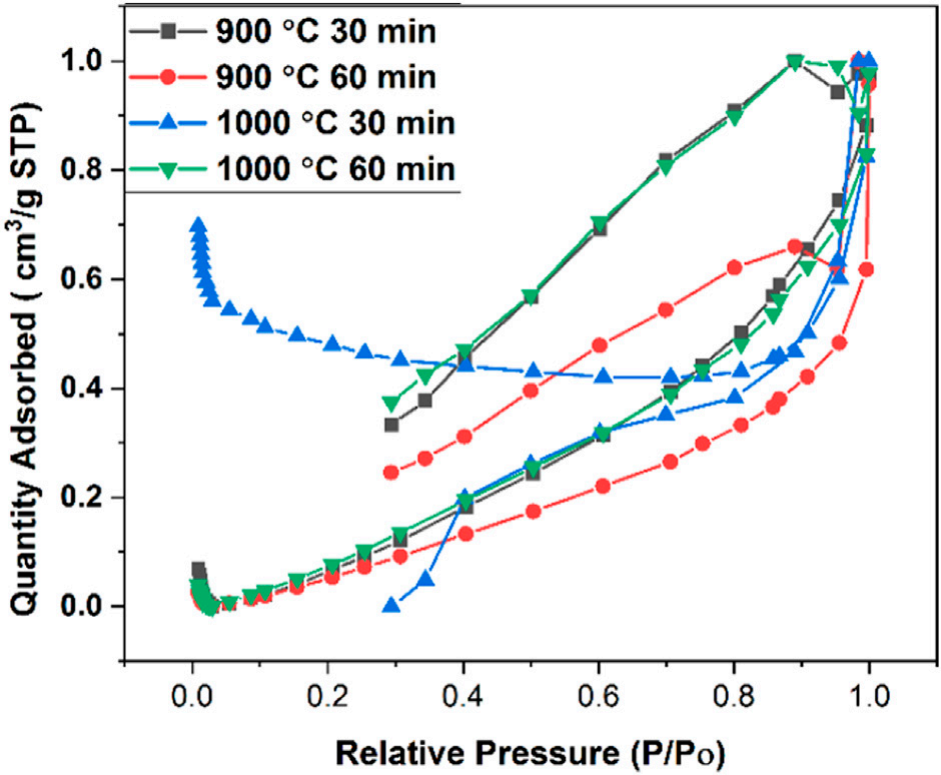
BET adsorption and desorption isotherm for calcined CFB.

**FIGURE 5:**
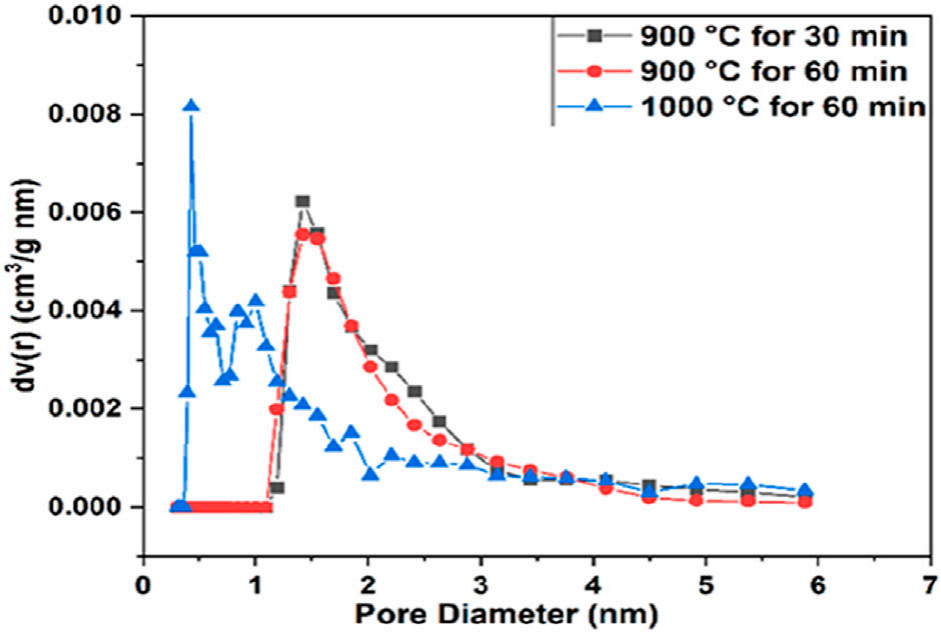
Pore size distribution of calcined CFBP.

**FIGURE 6:**
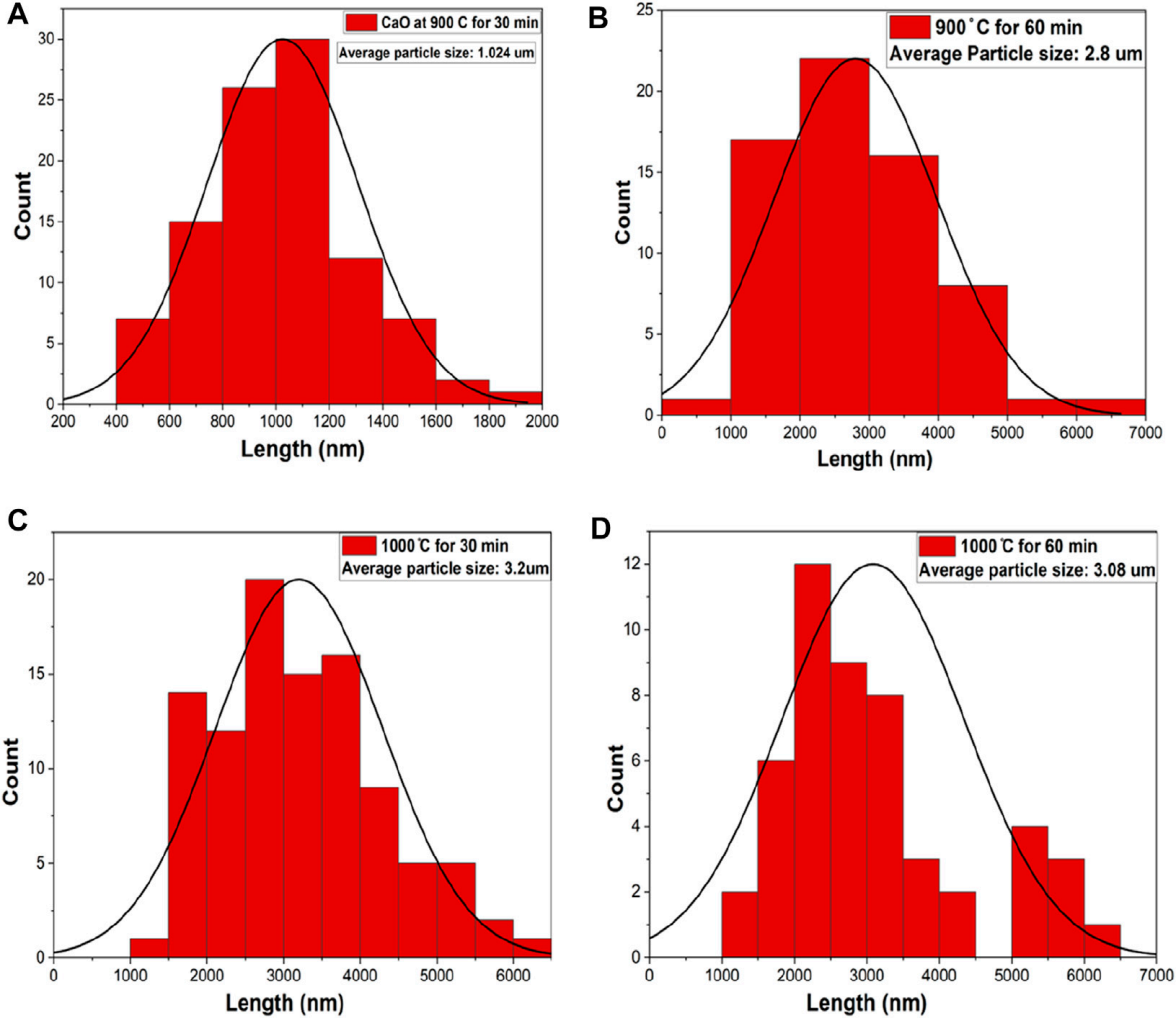
Particle size distribution of calcined CFB. **(A)** CFB calcined at 900°C for 0.5 h, **(B)** CFB calcined at 900°Cfor 1.0 h, **(C)** CFB calcined at 1000°C for 0.5 h, and **(D)** CFB calcined at 1000°C for 1.0 h.

The N_2_ adsorption–desorption isotherms of CaO from the pure cuttlefish bone are given in Figure 4 supplementary material. The synthesized CaO have a specific surface area mentioned in Table 1. The pore diameter of the calcium oxide is less than 2 nm, which means that the pores are in the range of microspores as shown in Figure 5. Particle size distributions were investigated because they may affect MB adsorption. Figure 6 shows the particle size of synthesized CaO at different temperatures for two different holding times. Particle size was calculated using ImageJ software. Figure 6A shows the normal distribution of CaO particles at 900°C for 30 min. The average particle size was 1.024 µm. The calcination temperature was kept constant while dwell time was increased, and this started deforming the individual particles, which agglomerated with other particles; hence, the average particle size was increased. By further increasing the temperature from 900°C to 1000°C, the particles started deforming, resulting in agglomeration. Particles started to deform and make a cluster with adjacent particles. Particles started to deform at higher temperatures, and hence particle size was increased.

**TABLE 1:** Surface area and pore diameter calculation for calcined CFB at 900°C for 30 min and 60 min and calcined CFB at 1000°C for 30 min and 60 min.

Sample	Specific surface area (m^2^ g^-1^)	Pore volume (cm^3^ g^-1^)	Pore diameter (nm)
CFB calcined at 900°C 0.5 h	2.6	7 × 10^−3^	1.4
CFB calcined at 900°C 1 h	1.7	9 × 10^−3^	1.4
CFB calcined at 1000°C 1 h	1.9	7 × 10^−3^	1.4

### 3.3 XRD analysis of CFBP and calcined CFBP

**FIGURE 7:**
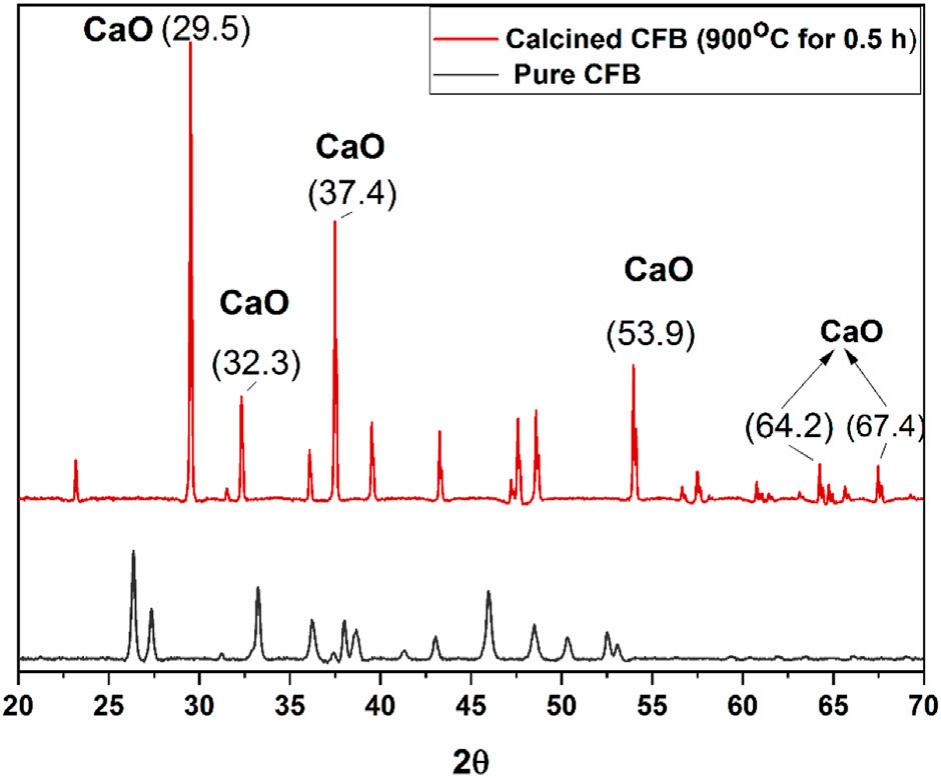
RD spectrum of CFB and calcined CFB at 900°C with a holding time of 0.5 h.

The X-ray diffraction of washed and dried CFBP and calcined CFBP at 900°C for 0.5 h dwell time is shown in Figure 7. The XRD spectrum of calcined CFBP at 900°C for 0.5 h shows the characteristic peaks of the CaO phase at 2θ values of 29.50, 32.22, 37.47, 53.95, 64.25, and 67.47. All these peaks of CaO were matched with the available literature (Lu et al., 2005; Nair et al., 2012). The main peak of the calcite phase (2θ = 26.58) was not detected in the XRD pattern of the calcined cuttlefish bone, thus confirming the complete decomposition of the CaCO_3_ phase into calcium oxide at 900°C 0.5 h. The presence of narrow and sharp peaks indicates that the calcined CFB is highly crystalline. Th XRD patterns of Khan et al. (2018)’s study resembled with hexagonal portlandite (Ca(OH)_2_) phase with space group P-3 m1 (Space Group No. 164, PDF Card No. 00–087–0673) as a major phase.

### 3.4 FTIR analysis of CFBP and calcined CFBP

**FIGURE 8:**
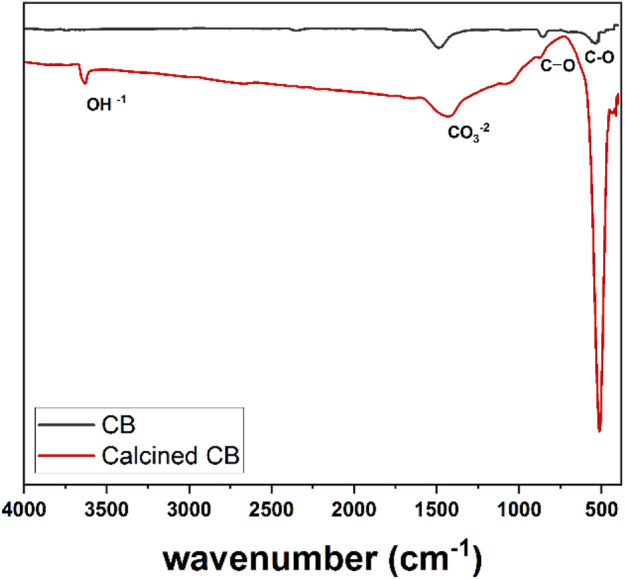
FTIR spectra of pure CFBP (referred to as CB in the picture) and calcined CFBP.

FTIR spectrometry was carried out to get insight into the functional groups found in the calcined CFBP. FTIR spectra of CFBP and calcined CFBP are reported in Figure 8. The spectra of calcined CFBP show a sharp vibrational band at 3600 cm^−1^ assigned to the stretching mode of the hydroxyl group OH^−1^ possibly related to the presence of Ca(OH)_2_. The presence of O-H IR stretch could be related to partial surface hydration after calcination due to moisture present in the environment. Both the samples exhibited similar IR bands assigned to the stretching mode of the (C–O) bond of carbonate between 1460–1520 cm^−1^. The weak band around 800 cm^−1^ is assigned to the bending mode of the C–O bond related to the partial carbonation of CaO nanoparticles. The strong peak at 500 cm^−1^ found for calcined material is assigned to the Ca–O bond stretching. The weak band registered at 2359 cm^−1^ is assigned to the unbounded atmospheric CO_2_ (Kalinkin et al., 2005). According to XRD and FTIR data, CaCO_3_ from cuttlefish bone was transformed into calcium oxide and some bits into calcium hydroxide due to partial hydrolysis of atmospheric moisture.

### 3.5 TGA and DTA analysis of raw CFBP

**FIGURE 9:**
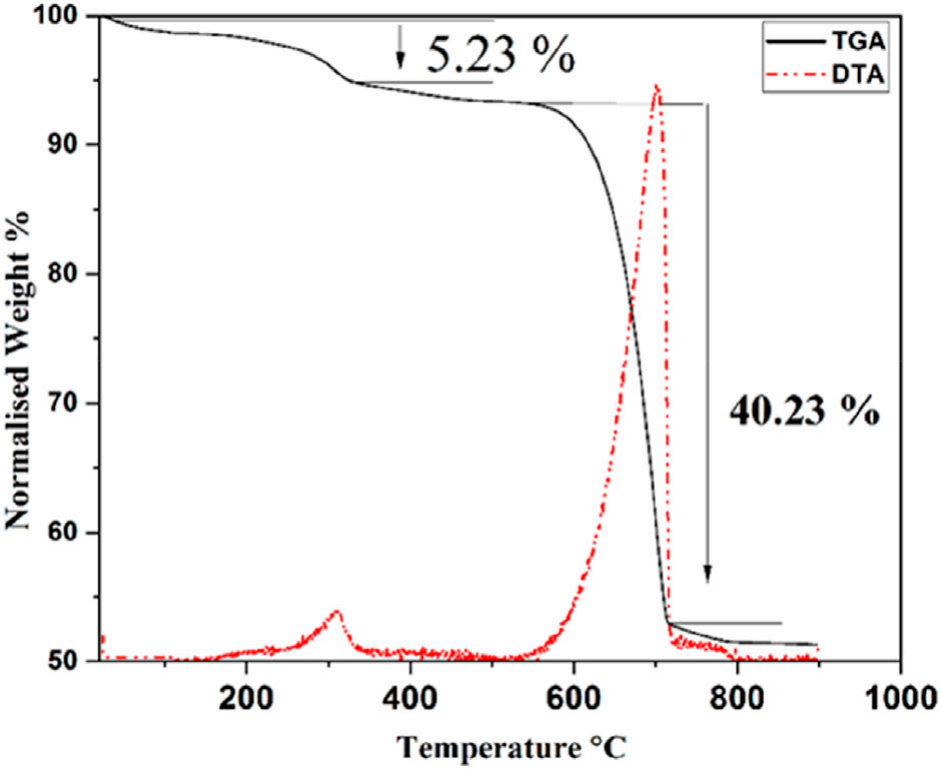
TGA and DTA curves of CFBP raw material.

TGA and DTA analysis results of CFB are shown in Figure 9. The thermal degradation of CFB was achieved in three steps. The first mass loss occurred at around 70–100°C, primarily due to moisture loss; the second weight-loss step, accounting for about 5.2 wt% loss, occurring between 280 and 340°C, is assigned to the decomposition of organic matter and proteins contained in the raw material; the third weight-loss stage, accounting for a 40.2 wt% loss, occurred between 580 and 720°C and is due to the decomposition of CaCO_3_ into calcium oxide (CaO) and carbon dioxide (CO_2_). The DTA curve clearly shows the devolatilization of organic material with a peak with a maximum of 310°C and a highly intense broad endothermic peak showing a maximum of around 720°C (Periasamy and Mohankumar, 2016).

### 3.6 Effect of CaO dose on the UV–Vis absorbance spectra of MB

**FIGURE 10:**
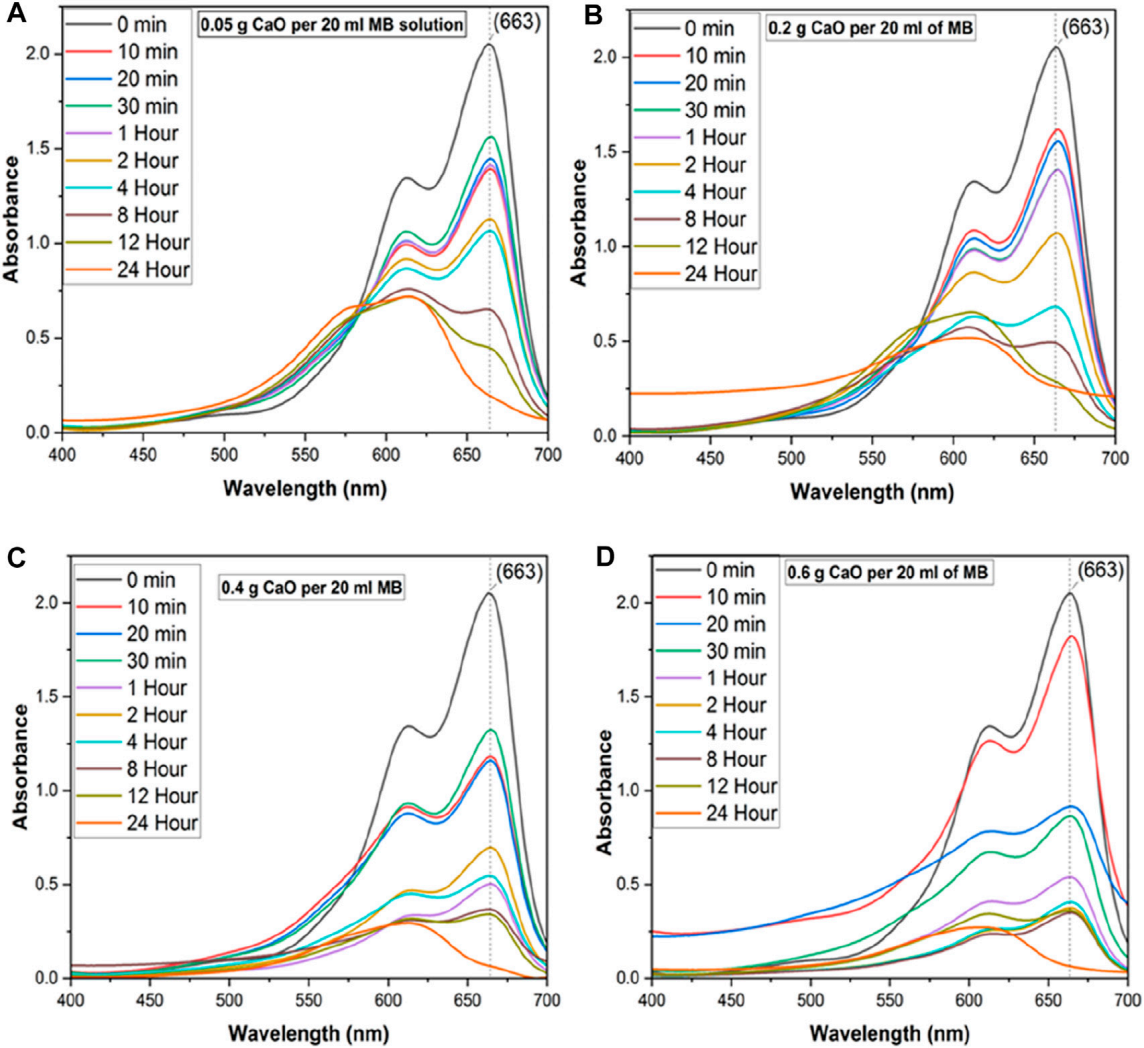
UV-Vis spectrum of MB on different CaO bio sorbent dosage for different contact time, **(A)** UV-Vis spectra of methylene blue with 0.05 g CaO/20ml MB solution up to 24 Hour time, **(B)** UV-Vis spectra of methylene blue with 0.2 g CaO/20 ml MB solution up to 24 h, **(C)** UV-Vis spectra of methylene blue with 0.4 g CaO /20 ml MB solution up to 24 h and **(D)** UV-Vis spectra of methylene blue with 0.6 g CaO/20 ml MB solution up to 24 h.

In this study, the efficiency of synthesized CaO was evaluated using MB dye as a model organic compound *via* the batch adsorption method. Figure 10 shows the absorbance curves of MB with 0.05 g CaO (A), 0.2 g CaO (B), 0.4 g CaO (C), and 0.6 g CaO (D) doses, respectively. The aforementioned CaO doses were added at the continuous stirring of 200 rpm for different periods, keeping the MB volume (20 ml) and concentration (10 mg/L) constant. It is shown in Figure 10 that 0.05 g (A) and 0.2 g (B) CaO doses had little effect on MB reduction. During the initial 1-h contact time (lines red–purple in the UV graph), the MB degradation was gradual. When the contact time was increased from 1 h to 2 h significant reduction in absorbance can be seen in the UV–Vis spectra of MB.

**FIGURE 11:**
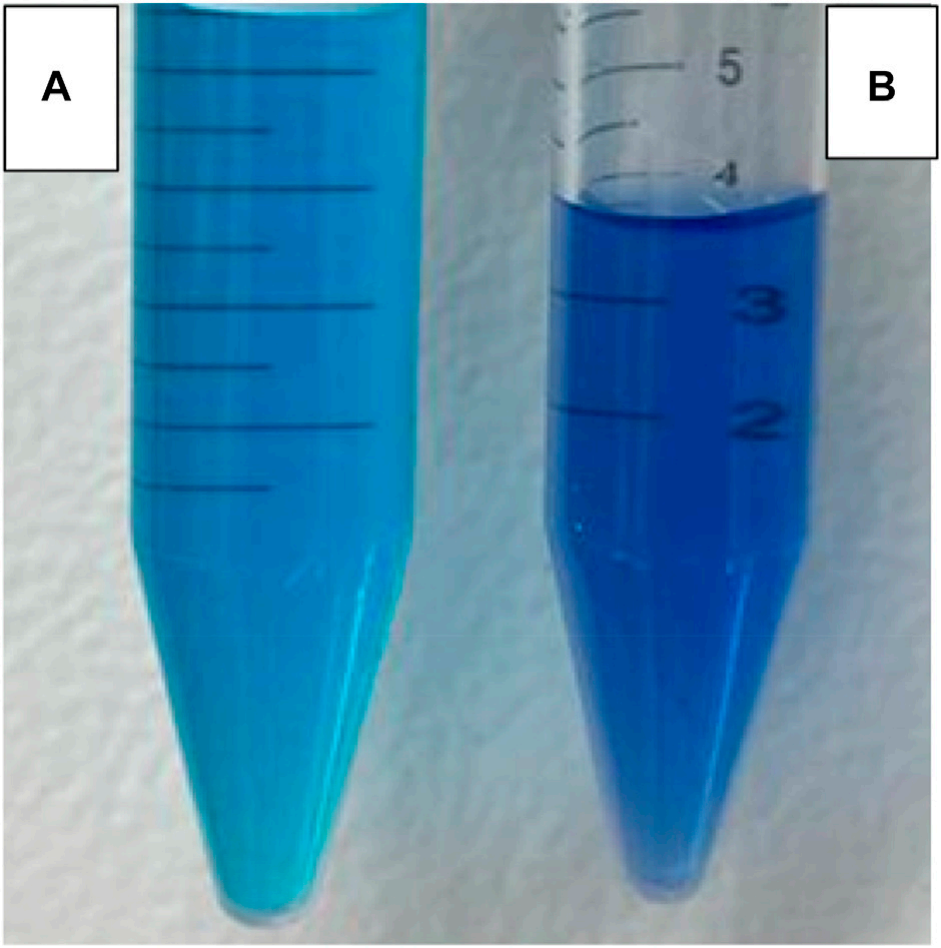
Change in MB color **(A)** without addition of CaO and **(B)** upon addition of 0.05 g CaO after 24 h.

With an increase in reaction time from 10 min, the significant peak of MB (*λ* 663) was reduced along with the broadening of the second MB at *λ* 613. When the time was increased from 2 h to 4 h, the second MB peak (*λ* = 613) started to become wider, and another peak (*λ* 560) appeared. This is called a hypochromic shift, a shift of a peak or signal to a shorter wavelength (higher energy), also called a blue shift peak, which could be due to MB precursor formation. This is a peak for Azure C dye, an MB precursor. When CaO was added to the MB solution, the pH of the MB solution became highly alkaline and increased up to 12.5 pH. Upon addition of CaO into MB, the solution color was changed from blue to a darker blue tone forming colored lipophilic species upon treatment with alkali, as shown in Figure 11.

Various literature records support the statement of MB peak shifting in an alkaline medium. Hameed et al. (2008) showed that MB reduction using pomelo peels was maximum at pH 10 because of the transition of the surface charge from the positive to negative regions that facilitates the adsorption of the cation (Dinh et al., 2019).

Mills et al. (2011) showed that MB hydrolysis occurs at pH 13 and MB is largely shifted to Bernthsen’s methylene violet (MVB). MVB has the λmax = 610, as shown in the literature. This feature arises because the pKa for the thiazine dyes decreases with decreasing degree of methylation, so that, for example, at pH 13, most (99%) of the thionine will be in its red, lipophilic deprotonated, free base form, whereas, for Azure B, at least 11.2% will still be in its purple–blue protonated form.

Lambda (*λ*) shifting of dyes is one of the most important parameters in photosynthesized galvanic cells. Mall et al. (2019) carried out a spectral study of photosensitizer dyes used in galvanic cells. They have shown that (Azure B and MB) have shown blue shifting from their monomer *λ* max in the presence of sodium dodecyl sulphate (SDS) in an alkaline medium. In this study, MB (664.0λ - 660.5λ) has shown blue shifting from their monomer *λ* max in the alkaline medium. The dye λ shifting proves the formation of a complex due to stronger electrostatic force between oppositely charged dye and adsorbent.

When the CaO dose was increased to 0.4 g (C) and 0.6 g (D), the MB peak reduced quickly in 10 min. For 0.4 g, peak broadening can be seen in Figure 10 from the start of the experiment, and it was increased at the maximum adsorption time, 24 h. At this time, the MB peak *λ* 663 diminished, and another broadened peak between 550–600 appeared. An increase in solution pH could be a reason for peak shifts in UV spectroscopic graphs, as pH is a significant factor that determines the material’s surface potential and could lead to MB hydrolysis. Zhang (2014) analyzed the degradation of MB by manganese oxide and found that at lower pH (<6.13), the surface charge of MnOx becomes positive owing to protonation. When the pH was higher than 6.13, the surface of MnOx was negatively charged, owing to a deprotonation reaction, which led to the formation of precursor between MB and MnOx by mutual attraction. When the time was increased from 2 h to 4 h, the second peak of MB (*λ* = 613) started to become wider, and another peak (*λ* 560) started to appear. When the time reached 24 h, the main MB peak around (*λ* 663) diminished mostly and produced precursors of a different wavelength. Using 0.05 g CaO dose, the peak shift occurred after a long contact time (12 h), but with 0.2 and 0.4 g CaO doses, it occurred after 8-h contact time, and for 0.6 g CaO dose, the peak shift started after 1–2-h contact time. By increasing the CaO dose, the number of available reactive sites increases. The second emerging peak of MB (*λ* 605) started to broaden up to *λ* 560, which belonged to the intermediates formed from MB degradation. The possible mechanism behind this peak shift has been discussed in the literature, which states that hydrolysis of MB leads mainly to the production of MVB as the main product of alkaline. The other major cationic thiazine dyes, which contain one or more amine-attached hydrogen atoms, also do not form hydroxy adducts but instead, are deprotonated in 0.1 M NaOH solution to very differently colored (λmax hypochromic shift in aqueous solution), red or orange lipophilic, free base forms of the original dye (Mills et al., 2011).

### 3.7 Effect of contact time on MB adsorption

**FIGURE 12:**
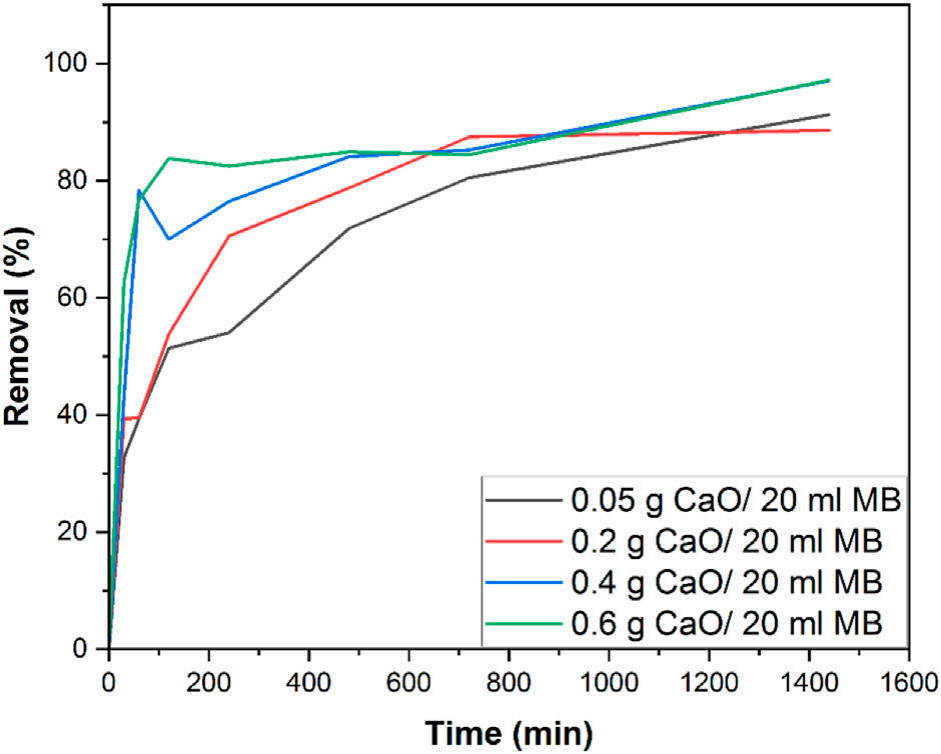
Removal % efficiency of 0.05 g, 0.2 g, 0.4 g, and 0.6 g calcined CFBP doses using 10 mg/L MB concentration.

The removal efficiency of MB was calculated using 0.05 g, 0.2 g, 0.4 g, and 0.6 g calcined CFBP (900°C for 0.5 h). The effect of contact time on the adsorption of MB dye was investigated using 10 mg/L MB concentration and varying the contact times from 10 min, 20 min, and 0.5–24 h, as shown in Figure 12. The effect of different CaO dosages 0.05–0.6 g/20 ml of MB was analyzed as a factor of time.

**FIGURE 13:**
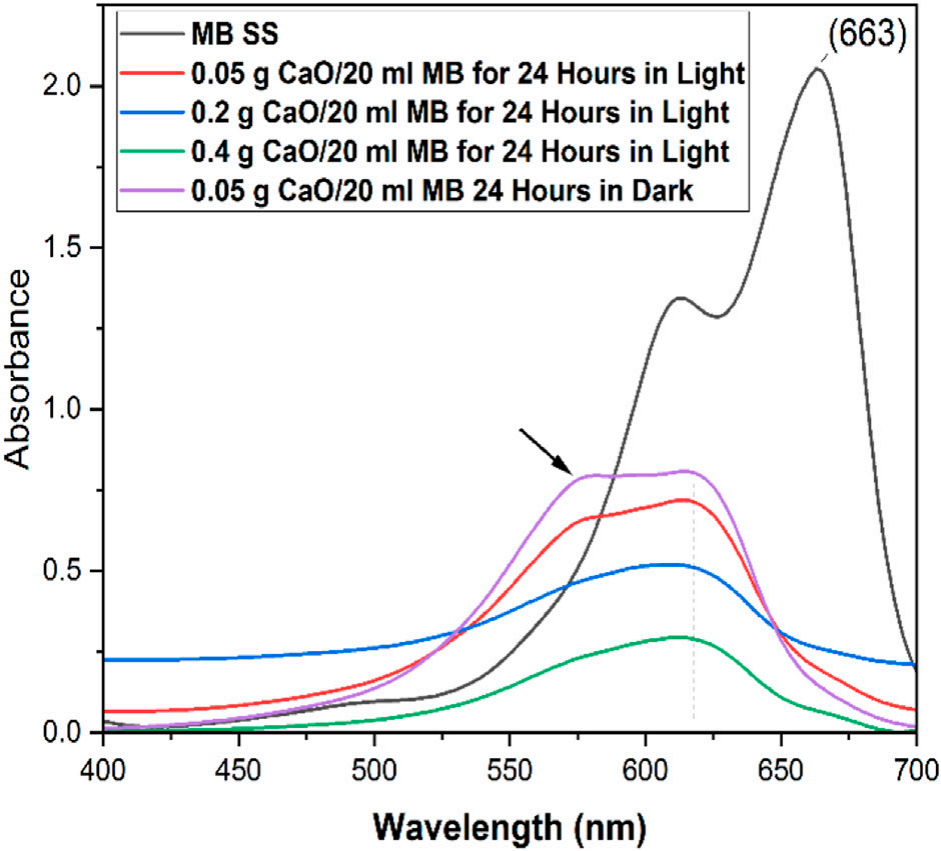
UV–Vis spectra of MB solution with different doses of CaO for 24 h in natural light and in dark.

It can be seen that a rapid increase in removal% was observed when the CaO dose was increased from 0.05 g to 0.4 g/20 ml MB in the initial 60 min contact time. As the contact time was increased from 60 min to 1400 min (24 h), a gradual increase in removal% was observed. It was observed that the rate of removal increased up to 0.4 g CaO. At 0.6 g, the removal rate seemed constant and almost equal to 0.4 g CaO. The profile showed that maximum adsorption efficiency (98% removal efficiency) was achieved by 0.4 g CaO dose and was similar for 0.6 g CaO/20 ml MB in the total 24 h contact time. This study was carried out under natural room light, and many studies have used CaO as a photocatalytic material. However, in this study, we found that CaO did not work photocatalytically. To prove this statement, we tested our synthesized CaO with a minimum dose of 0.05 g for 24 h in a completely dark environment. The UV visible spectrum in Figure 13 shows no great difference between a sample kept in the total dark for 24 h and one which was kept under natural light for 24 h. This means the synthesized CaO from CFB calcination is not a photocatalytic material. The peak shift occurred at *λ* 605, and intermediates were formed.

As calcined CFB powder (900°C for 0.5 h) is mainly CaO which becomes Ca(OH)_2_ when it reacts with water. The reported band gap of Ca(OH)_2_ is 5.7 eV. These band gaps suggest photocatalytic activity under visible light conditions is hardly possible. However, Song et al. (2015) suggested that the measured band gap for Ca(OH)_2_ is further decreased to 3.2 eV by removing the energy gap between the bottom of the conduction band and the Fermi level (2.5 eV) from the band gap of conduction and valence (5.7 eV). This bandgap provides an excitation wavelength of roughly 400 nm, allowing the catalyst to function near the edge of visible light. MB’s absorbance bands at 665 nm and 615 nm correspond to monomer and dimer units, respectively. Those two compounds have been identified in the literature as Azure B and Azure C, respectively. These intermediates, especially Azure B, possess pharmacological effects such as inhibiting peptide aggregation and endo-toxic shocks (Petzer et al., 2012).

There are various sources available for calcium-based adsorbent synthesis. Table 2 provides an overview of CaO-based adsorbents, their synthesis conditions, and their maximum removal efficiency. This study shows that CaO synthesized from CFB showed equal removal efficiency to other CaO-based adsorbents, using low calcination temperature with a low holding time. Isothermal adsorption models are often applied to describe the adsorption behavior between two phases in solution, which are very important to optimize the adsorption effect. Adsorption isotherm studies help in understanding molecular distribution between liquid and solid phases when reaching adsorption equilibrium (Azizian, 2004). The Langmuir and Freundlich isothermal models investigate the affinity effect between the adsorbent surface and MB (Vasanth Kumar and Sivanesan, 2006). The Langmuir isothermal model (Eq. 5–6) is always used to describe the monolayer adsorption on the adsorbent surface. In contrast, the Freundlich isothermal model (Eq. 7) shows that it exists in the form of multilayer adsorption. The Langmuir equation is valid for a homogeneous surface. The linearized form of the Langmuir adsorption isotherm model is given in Eq. 5. For this work, adsorption studies were carried out using Langmuir isotherm and Freundlich isotherm (Chung et al., 2015). The Langmuir isothermal model (Eq. 5) is always used to describe the monolayer adsorption on the adsorbent surface. In contrast, the Freundlich isothermal model (Eq. 7) shows that it exists in the form of multilayer adsorption. The Langmuir equation is valid for a homogeneous surface. The linearized form of the Langmuir adsorption isotherm model is given in Eq. 5. For this work, adsorption studies were carried out using Langmuir isotherm and Freundlich isotherm (Chung et al., 2015).

**TABLE 2:** Comparison of different CaO-based adsorbents and their removal efficiency.

Adsorbent	Resource	Experimental conditions	Adsorbate	Removal (%)	References
CaO	Activated sludge	Adsorption time of 90 min, CaO-SA dosage of 1 g/L, pH of 5, and adsorption temperature of 40°C	Cd(ii)	99.74%	Gu et al. (2021)
CaO	Chicken eggshell	900°C for 6 h	MB	98% in 180 min	Jaiswal et al. (2021)
CaO	Hen eggshell	Sol-gel	Pb(II)	99.07% at an initial concentration of 75.46 ppm, pH of 6.94, adsorbent dose of 0.838 g, and contact time of 101.97 min	Jalu et al. (2021)
CaO	Crab shell	Calcination at 900°C	Naphthalene solution	97.54% (5 mg/L) to 95.08% (10 mg/L) with interaction duration enhancing from 20 min to 30 min	Kumar et al. (2022)
Nano CaO	Gastropod shell	Sol-gel	Cr (VI)	Sorption capacity (qm, mg/g) value of 125 mg/g	Oladoja et al. (2012)
(Ca(OH)_2_)	Clam shells	Calcined at 900°C for 2 h	Methyl red dye	Catalyst loading of 3 g/L showed that at a concentration of 20 mg/L of MR, complete degradation was observed after 35 min of treatment	Narayan et al. (2018)
CaO	CFB	Calcined at 900°C for 0.5 h	MB dye	98% with 0.4 g CaO/20 ml of MB dye in 24 h	This study

### 3.8 Langmuir isotherm

**FIGURE 14:**
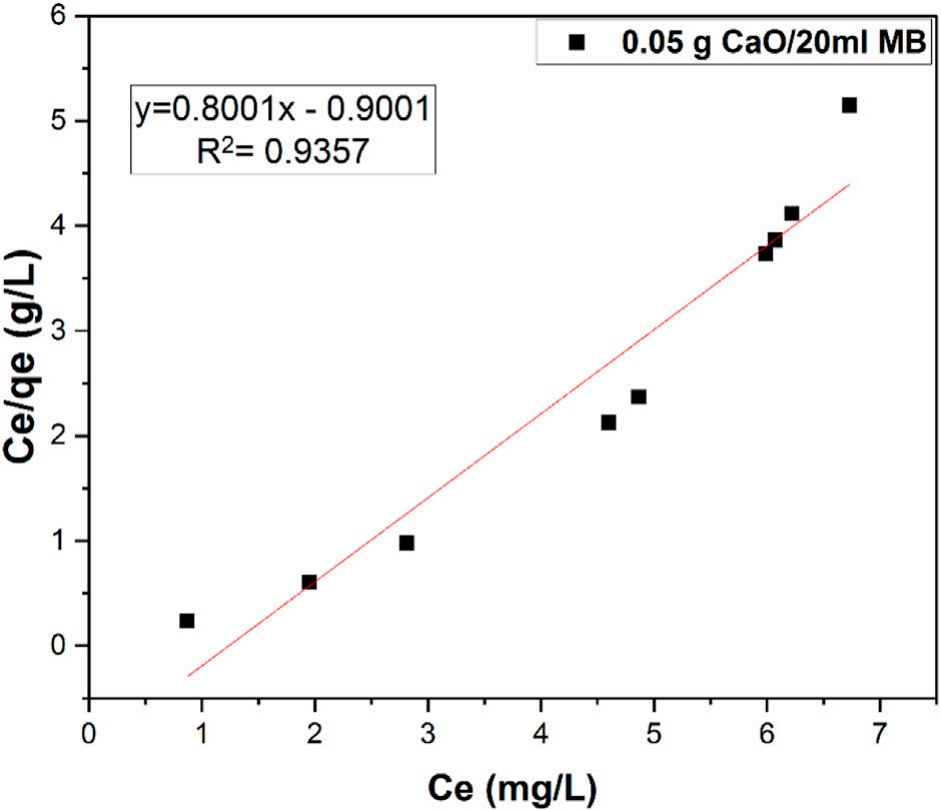
Langmuir isotherm studies for 0.05 g CaO adsorbent dose per 20 ml MB (10 mg/L).

$$\frac{Ce}{qe}=\frac{1}{q_{m}b}+\frac{Ce}{Q_{m}},$$ (5)

where qe (mg/g) is the amount adsorbed at equilibrium, Ce (mg/L) is the equilibrium concentration, qm is the Langmuir constant representing maximum monolayer adsorption capacity, and b is the Langmuir constant related to the energy of adsorption. The essential characteristics of the Langmuir isotherm can be expressed as the dimensionless constant R_L_.

$$R_{L}=\frac{1}{1+bC_{o}},$$ (6)

where R_L_ is the equilibrium constant that indicates the type of adsorption, b is the Langmuir constant, and Co is the various concentration of CaO solution. The R_L_ values between 0 and 1 indicate favorable adsorption (Ganesan et al., 2013). Figure 14 shows the linearized form of Langmuir isotherm using 0.05 g synthesized CaO per 20 ml of MB, which gives the best fit of data compared with the Freundlich plot. The Langmuir adsorption isotherm is used to describe the equilibrium between the adsorbate and adsorbent system, where the adsorbate adsorption is limited to one molecular layer at or before a relative pressure of unity is reached, although the isotherm initially proposed by Langmuir in 1918 is generally suitable for describing the chemisorption process when ionic or covalent chemical bonds are formed between the adsorbent and the adsorbate.

### 3.9 Freundlich isotherm

The Freundlich adsorption isotherm model includes considerations of surface heterogeneity and an exponential distribution of the active sites and their energies. The isotherm is adopted to describe reversible adsorption and is not restricted to monolayer formation. The linearized in logarithmic form and the Freundlich constants can be expressed as

ln⁡qe=lnkf+nlnCe, (7)

where qe = adsorbed amount of adsorbent at equilibrium (mg/g), Ce = equilibrium concentration of adsorbate, kf = adsorption capacity (mg/g), and n = heterogeneity factor (dimensionless). Here, kf and n are Freundlich constants which represent adsorption capacity and adsorption intensity, respectively, and their values can be derived from the intercept and slope of the linear graph of log qe versus log Ce.

**FIGURE 15:**
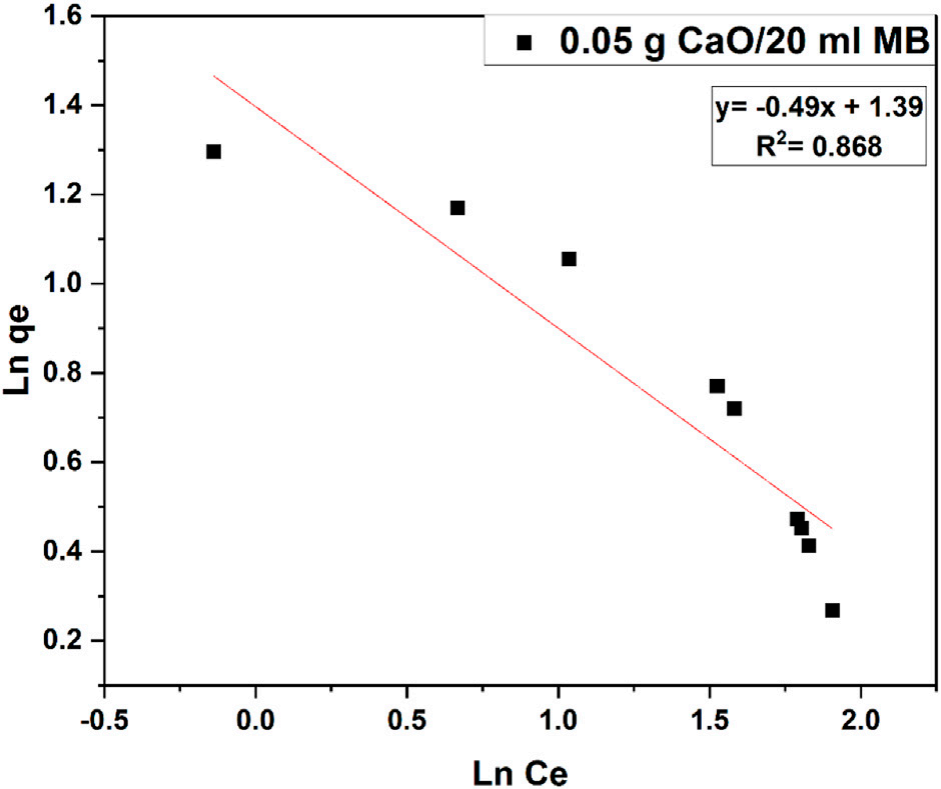
Freundlich isotherm studies for 0.05 g CaO adsorbent dose per 20 ml MB (10 mg/L).

Also, Freundlich isotherm was studied on the same sample data as shown in Figure 15, but the data seemed to fit with Langmuir isotherm as the R^2^ value of Langmuir is much higher than that of Freundlich isotherm.

### 3.10 Methylene blue kinetics on 0.05 g CaO biosorbent dose

To investigate the mechanisms of the adsorption process, two different kinetic models, namely, the first- and second-order Lagergren models, were applied to describe the kinetics of the MB adsorption onto calcium oxide. The best-fit model was selected according to the linear regression correlation coefficient values, R^2^ (Johnson, 1990). The kinetic behavior of dye degradation was studied using first-order and second-order Lagergren models on 0.05 g CaO dose. The model equations are given in the following.

#### 3.10.1 First-order kinetic model

$$\frac{dq_{t}}{d_{t}}=k_{1}\left(q_{e}-q_{t}\right),$$ (8)

where q_e_ (mg/g) and q_t_ (mg/g) are the amounts of MB adsorbed on the adsorbent at equilibrium and at any time t (min), respectively, and k_1_ (min^−1^) is the rate constant of the first-order model. The integrated form of the above equation with the boundary conditions t = 0 to >0 (q = 0 to >0) is rearranged to obtain the following time-dependence function:

$$\log\left(qe-qt\right)=\log\left(qe\right)-\frac{k_{1}t}{2.303}.$$ (9)

**FIGURE 16:**
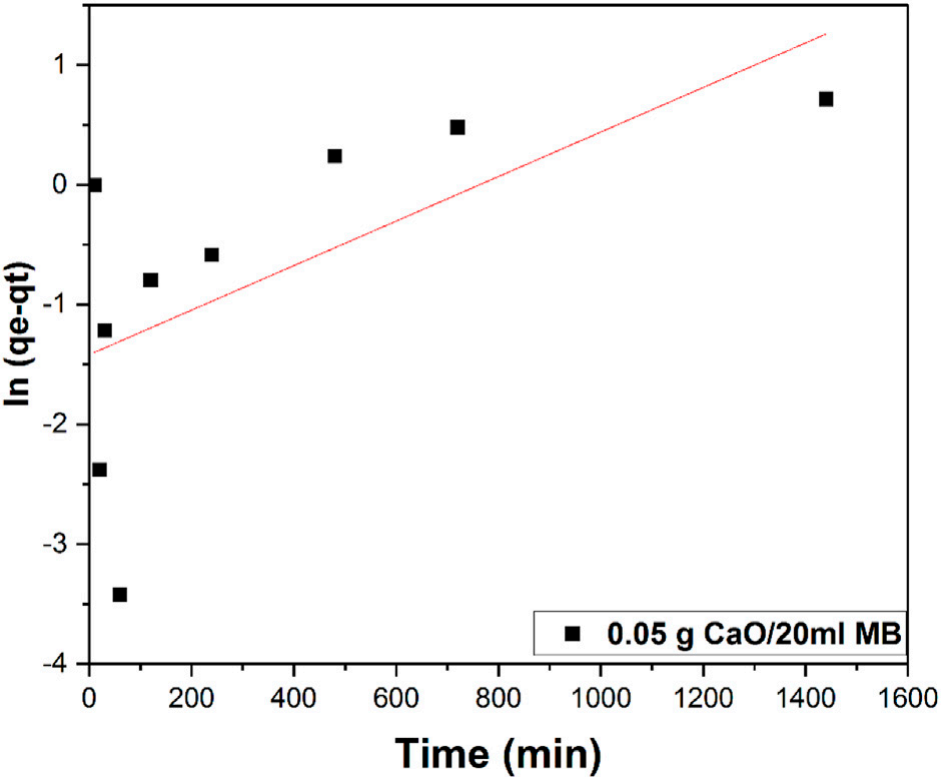
First-order kinetic model plot of 0.05 g/20 ml MB concentrations.

The plot ln(qe-qt) versus time (t) as shown in Figure 16 does not fit with our data. The first-order model considers the rate of occupation of the adsorption sites proportional to the number of unoccupied sites.

#### 3.10.2 Second-order kinetic model

The second-order kinetic model is expressed as

$$\frac{d_{q}}{d_{t}}=k_{2}{\left(q_{e}-q_{t}\right)}^{2}.$$ (10)

Eq. 10 can be rearranged and linearized as

$$\frac{t}{q_{t}}=\frac{1}{k_{2}q_{e^{2}}}+\frac{t}{q_{e}}.$$ (11)

**FIGURE 17:**
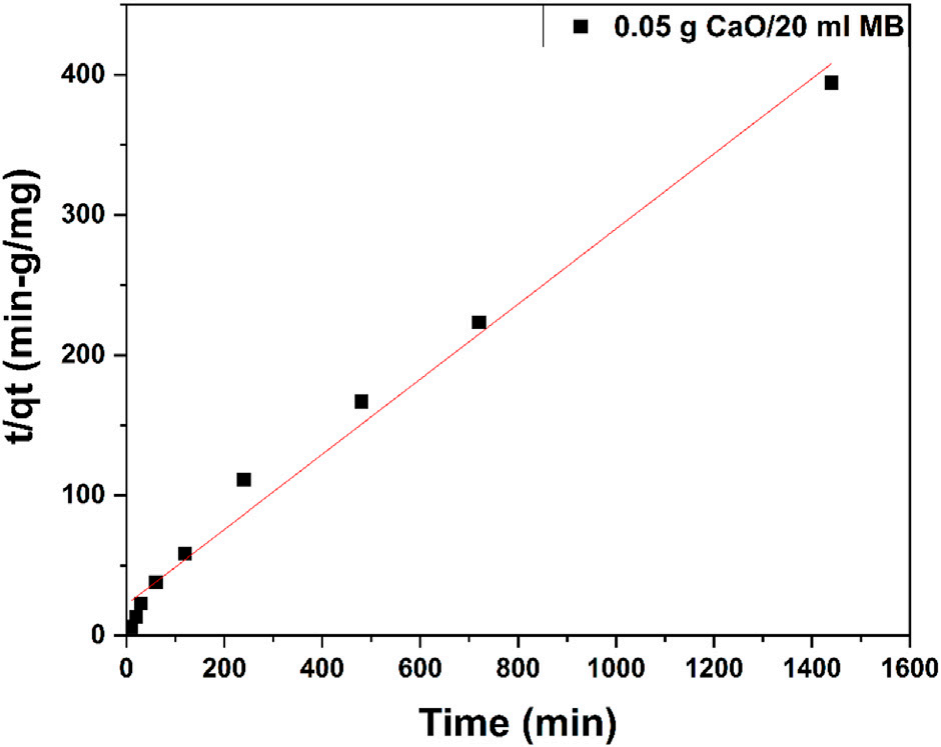
Second-order kinetic model plot of 0.05 g/20 ml MB concentrations.

The plot t/qt versus time (t) Figure 17 shows a straight line. The second-order kinetic values of qe and k_2_ were calculated from the slope and intercept of the plots t/qt versus t. Table 3 depicts the computed results obtained from the second-order kinetic model. The calculated qe values agree well with the experimental qe values for second-order kinetics with high regression values.

**TABLE 3:** Constant parameters and correlation co-efficient calculated for Langmuir and Freundlich adsorption isotherm models at room temperature for MB adsorption at 10 (mgL^−1^) at room temperature.

Adsorption isotherms			Constants	
Langmuir	Qm (mg.g^-1^)	K_L_	R_L_	R^2^
	1.25	1	0.1	0.93
Freundlich	Kf (mg.g^-1^) 4.04	n(Lmg^−1^) −2.04		R^2^ 0.87

Kinetic modes of calcined CFBP (900°C for 0.5 h) are shown in Figure 17. When correlation coefficient values R^2^ of both orders were compared, the R^2^ value of the second-order was higher than the first-order as shown in Table 4. Thus, the pseudo-second-order kinetic model fits best with 0.05 g calcined CaO per 20 ml of MB since chemisorption may govern adsorption, which is also proved by Langmuir isotherm studies.

**TABLE 4:** Summary of kinetic studies for MB blue using 0.05 g CaO per 20 ml MB.

Concentration (mg/L)	First-order adsorption			Second-order adsorption		
10	qe (mg.g^-1^) 0.25	k_1_ (min.mg^-1^) 1.29E-06	R^2^ 0.32	qe (mg.g^-1^) 3.73	K_2_ 0.0033	R^2^ 0.98

## 4 Conclusion

In this study, a calcination technique was used to synthesize highly ordered CaO as an effective adsorbent representing a cheaper, greener, and sustainable alternative solution for organic-polluted water remediation. The CaO synthesis was optimized by varying the calcination temperature and holding time to analyze the morphological changes. SEM analysis proved the formation of highly ordered cubicle-structured CaO at 900°C for 0.5 h. FTIR and XRD analyses showed the presence of Ca–O bonds stretching vibrations. Furthermore, 10 mg/L MB standard concentration was used to assess CaO adsorption properties. As CaO is highly alkaline, it was noticed that while adding it into the solution, the pH was increased up to (12.5). The pH of a solution is a significant factor that determines the material’s surface potential and could lead to MB hydrolysis and can produce precursors. For this work, the MB solution was also shifted to a lower wavelength, and the solution color was changed to a purplish tone, which proves the formation of the MB precursor. Furthermore, two different adsorption isotherms were used, and the Langmuir isotherm model fit the data well, giving R^2^ 0.93, which demonstrates that chemisorption with a monolayer adsorption mechanism is prevalent during the adsorption of MB by calcined CFB (900°C for 0.5 h). To understand the adsorption rate, pseudo-first-order and pseudo-second-order kinetic rate equations were also studied for CaO, and pseudo-second-order fit the data well, giving R^2^ 0.98, which suggests that MB is chemisorbed. These results indicate the technological advantages making CaO potentially suitable for adsorption material. The dye precursors formed, especially Azure B, have pharmacological effects such as inhibiting peptide aggregation and endo-toxic shocks.

## Data Availability

The raw data supporting the conclusions of this article will be made available by the authors, without undue reservation.
